# Supplementary material for: Individual variability of neural computations underlying flexible decisions
Source: Nature. 2024 Nov 28;639(8054):421–9. doi: 10.1038/s41586-024-08433-6 (PMC11903320; doi:10.1038/s41586-024-08433-6)
Supplement: Supplementary file 2 — Reporting Summary [file 41586_2024_8433_MOESM2_ESM.pdf]

nature portfolio

Corresponding author(s): Carlos Brody, Marino Pagan

Last updated by author(s): Oct 10, 2024

Reporting Summary

Nature Portfolio wishes to improve the reproducibility of the work that we publish. This form provides structure for consistency and transparency in reporting. For further information on Nature Portfolio policies, see our Editorial Policies and the Editorial Policy Checklist.

Statistics

For all statistical analyses, confirm that the following items are present in the figure legend, table legend, main text, or Methods section.

n/a	Confirmed	
☐	☒	The exact sample size (*n*) for each experimental group/condition, given as a discrete number and unit of measurement
☐	☒	A statement on whether measurements were taken from distinct samples or whether the same sample was measured repeatedly
☐	☒	The statistical test(s) used AND whether they are one- or two-sided<br>*Only common tests should be described solely by name; describe more complex techniques in the Methods section.*
☐	☒	A description of all covariates tested
☐	☒	A description of any assumptions or corrections, such as tests of normality and adjustment for multiple comparisons
☐	☒	A full description of the statistical parameters including central tendency (e.g. means) or other basic estimates (e.g. regression coefficient) AND variation (e.g. standard deviation) or associated estimates of uncertainty (e.g. confidence intervals)
☐	☒	For null hypothesis testing, the test statistic (e.g. *F*, *t*, *r*) with confidence intervals, effect sizes, degrees of freedom and *P* value noted<br>*Give P values as exact values whenever suitable.*
☒	☐	For Bayesian analysis, information on the choice of priors and Markov chain Monte Carlo settings
☒	☐	For hierarchical and complex designs, identification of the appropriate level for tests and full reporting of outcomes
☒	☐	Estimates of effect sizes (e.g. Cohen's *d*, Pearson's *r*), indicating how they were calculated

Our web collection on statistics for biologists contains articles on many of the points above.

Software and code

Policy information about availability of computer code

Data collection	All code for data collection was written in Matlab 2019b. The code for behavioral training and behavioral data collection is available at https://github.com/Brody-Lab/flexible_decision_making_training .
Data analysis	Spike sorting was performed using MountainSort version 3, available at https://github.com/flatironinstitute/mountainsort . Custom code for training, analysis, and engineering of RNNs is available at: https://github.com/Brody-Lab/flexible_decision_making_rnn. Custom code for the analysis of neural data and behavior is available at: https://github.com/Brody-Lab/flexible_decision_making_rats. Recurrent neural networks were trained using the Adam optimizer and implemented in the Python JAX framework.

For manuscripts utilizing custom algorithms or software that are central to the research but not yet described in published literature, software must be made available to editors and reviewers. We strongly encourage code deposition in a community repository (e.g. GitHub). See the Nature Portfolio guidelines for submitting code & software for further information.

Data

Policy information about availability of data

All manuscripts must include a data availability statement. This statement should provide the following information, where applicable:

- Accession codes, unique identifiers, or web links for publicly available datasets
- A description of any restrictions on data availability
- For clinical datasets or third party data, please ensure that the statement adheres to our policy

The rat behavioral and electrophysiological data are available at: https://github.com/Brody-Lab/flexible_decision_making_rats . Modeling data are available at: https://github.com/Brody-Lab/flexible_decision_making_rnn .

Human research participants

Policy information about studies involving human research participants and Sex and Gender in Research.

Reporting on sex and gender	N/A
Population characteristics	N/A
Recruitment	N/A
Ethics oversight	N/A

Note that full information on the approval of the study protocol must also be provided in the manuscript.

Field-specific reporting

Please select the one below that is the best fit for your research. If you are not sure, read the appropriate sections before making your selection.

☒ Life sciences ☐ Behavioural & social sciences ☐ Ecological, evolutionary & environmental sciences

For a reference copy of the document with all sections, see nature.com/documents/nr-reporting-summary-flat.pdf

Life sciences study design

All studies must disclose on these points even when the disclosure is negative.

Sample size	No statistical methods were used to predetermine sample sizes, and sample sizes were determined by the limits of what data could be collected within a reasonable time frame and standards of the field (see e.g. Hanks et al., 2015; Duan et al., 2021).<br>A total of 26 rats were used for the experiments presented in this study. Of these, 7 rats were used for electrophysiology recordings, and 3 rats were implanted with optical fibers for optogenetic inactivation.
Data exclusions	No data were excluded from the analyses. Analysis of behavior was performed only for rats that performed at least 120,000 valid trials, i.e. where the rat maintained fixation for the full duration of the pulse train before making a decision.
Replication	Findings about the overall trial-averaged neural dynamics were replicated across rats (see Extended Data Fig. 7). Findings about the context-dependent integration dynamics were replicated for individual rats across half-split experimental sessions (see Extended Data Fig. 4).
Randomization	All subjects were randomly allocated into experimental groups.
Blinding	All behavioral and neural responses in our experiments were objectively measured by automated hardware and software system that do not require human intervention, and therefore were blinded to investigators.

Reporting for specific materials, systems and methods

We require information from authors about some types of materials, experimental systems and methods used in many studies. Here, indicate whether each material, system or method listed is relevant to your study. If you are not sure if a list item applies to your research, read the appropriate section before selecting a response.

Materials & experimental systems

n/a	Involved in the study
☒	☐ Antibodies
☒	☐ Eukaryotic cell lines
☒	☐ Palaeontology and archaeology
☐	☒ Animals and other organisms
☒	☐ Clinical data
☒	☐ Dual use research of concern

Methods

n/a	Involved in the study
☒	☐ ChIP-seq
☒	☐ Flow cytometry
☒	☐ MRI-based neuroimaging

Animals and other research organisms

Policy information about studies involving animals; ARRIVE guidelines recommended for reporting animal research, and Sex and Gender in Research

Laboratory animals	Long–Evans rats (Rattus norvegicus) between the ages of 6 and 24 months.
Wild animals	No wild animals were used in the study.
Reporting on sex	Only male rats were used in our experiments, for two reasons: 1) Our study focuses on individual variability of the neural mechanisms of cognition. Because we expect to find large differences across sexes, and in females also across different phases of the estrous cycle (see e.g. Clemens et al., Current Biology, 2019), including both male and female rats would have required a much larger sample size. 2) Our high-throughput behavioral training pipeline, which was necessary to collect a sufficient amount of data across multiple individuals, relies on rats sharing behavioral rigs and spaces. Our previous experience indicates that it is difficult to train male rats to perform complex cognitive tasks when they are exposed to the scent of female rats, with also a significant increase in aggressive behavior.
Field-collected samples	No field-collected samples were used in the study.
Ethics oversight	All animal use procedures were approved by the Princeton University Institutional Animal Care and Use Committee (IACUC).

Note that full information on the approval of the study protocol must also be provided in the manuscript.
